# Editorial: Postural control priorities and effective motor learning: volume II

**DOI:** 10.3389/fnhum.2026.1974219

**Published:** 2026-09-23

**Authors:** Tadayoshi Asaka, Naoya Hasegawa, Yoshiro Okubo

**Affiliations:** 1 Hokkaido University, Sapporo, Japan; 2 Faculty of Health Sciences, Hokkaido University, Sapporo, Japan; 3 Neuroscience Research Australia, Sydney, NSW, Australia

**Keywords:** aging, cognitive load, motor development, motor learning, neurological diseases, postural control, sensory biofeedback, sensory weighting

## 1 Introduction

Human postural control, defined as the ability to maintain or restore postural balance in any position or during any motor activity, is a highly sophisticated, goal-directed process requiring continuous sensorimotor integration. It relies on the central nervous system (CNS) to dynamically reweight multisensory afferents such as visual, vestibular, and somatosensory, refining sensorimotor estimates to preserve upright stability amidst varying task constraints. Previous studies have elucidated how the CNS acquires and adapts postural control strategies. However, understanding these control strategies becomes challenging when competing postural priorities exist, such as trade-offs between stability and flexibility, automatic vs. voluntary control, and inherent vs. compensatory mechanisms. The relative weighting of these sensory inputs depends on environmental demands and behavioral goals. Gaining a better understanding of these priorities can aid in establishing effective, personalized approaches to motor learning in healthy younger and older adults, improving rehabilitation for individuals with neurological diseases, and promoting healthy daily living.

This Research Topic aims to consolidate current knowledge and foster new research into the optimization of postural control mechanisms. Moving beyond traditional views that emphasize the automatic regulation of balance, this Research Topic, “*Postural control priorities and effective motor learning: volume II*,” brings together seven outstanding articles that delve into the supraspinal regulation and adaptive prioritization of posture. Our goal is to create a comprehensive resource that illuminates the underpinnings of postural control priorities and their implications for effective motor learning. By dissecting the nuances of how different sensory inputs influence postural stability, this volume highlights how the CNS dynamically modulates its functional priorities based on developmental stages, cognitive demands, aging, and neurological deficits, thereby guiding future interventions and educational strategies tailored for both healthy individuals and those recovering from neurological conditions.

## 2 An overview of published articles

### 2.1 Developmental and cognitive priorities in postural control

The maturation of postural control strategies is particularly evident during childhood. Valtr et al. investigated phase-specific postural adjustments during challenging balance tasks, highlighting how children and adults differ in prioritizing dynamic stability and segment coordination. Furthermore, cognitive capacity strongly influences motor priorities. Christ et al. explored postural strategies in children with Attention-Deficit/Hyperactivity Disorder (ADHD). Their findings suggest that attentional demands may differentially influence postural strategies in children with ADHD whose attentional resources are characteristically constrained, distinct static and dynamic balance profiles emerge, demonstrating that cognitive load significantly alters the hierarchical prioritization of motor strategies during development.

### 2.2 Visuomotor priorities and sensory reweighting in aging

As individuals age, sensory acuity declines, requiring the CNS to reweight sensory inputs and reprioritize how stability is maintained. Rasku et al. provided a comprehensive analysis of sex and age differences in postural control among community-dwelling older adults. Their work emphasizes that baseline mechanisms of static postural stability shift distinctly across demographics, highlighting the necessity of age- and sex-specific normative data when evaluating postural control. Strikingly, Sakazaki et al. demonstrated how prioritization of visual information may override biomechanical safety. They found an age-independent visuomotor effect wherein near-gaze fixation promotes the use of “spin turns” during walking—a strategy that is biomechanically less stable than step turns. Importantly, for older adults, this greater reliance on immediate visual information may contribute to maladaptive behaviors associated with increased fall risk, highlighting a potential vulnerability in age-related postural control.

### 2.3 Restoring priorities: clinical assessments and motor adaptation

When disease or injury disrupts the CNS, therapeutic interventions must focus on safely reorganizing postural priorities. Brodsky et al. contributed a vital narrative review on using perturbation-based modeling to assess balance control mechanisms. By mathematically modeling how patient populations reweigh sensory feedback during perturbations, clinicians can objectively identify which sensory priorities have been compromised. For stroke survivors, Kuipers et al. provided a scoping review on modifying the Center of Pressure (CoP) to reduce fall risks, effectively mapping out strategies to modify or restore impaired postural control mechanisms. Finally, focusing on proactive motor adaptation, Tomita et al. demonstrated that visual feedback adaptation significantly enhances arm-posture coordination during floor-surface perturbations. This suggests that targeted visual feedback can actively retrain the CNS to improve efficient inter-limb coordination during unexpected losses of balance.

Collectively, these seven studies illustrate a vital shift in the field: moving from merely identifying postural deficits to elucidating the underlying sensory, biomechanical, and cognitive priorities. By spanning a wide demographic spectrum—from early development and neurodivergence to aging and neurological rehabilitation—this volume emphasizes that human balance cannot be reduced to generalized models. Instead, these articles advocate for highly context-specific assessments that account for cognitive load, visual reliance, and individual biomechanics. Ultimately, this Research Topic bridges the gap between fundamental physiological research and translational clinical practice, laying the groundwork for next-generation, individualized rehabilitation strategies.

## 3 Future perspectives

Looking forward, the insights gathered in this Research Topic illuminate three important directions for future research in postural control and motor learning. First, there is a critical need to advance personalized, lifespan-oriented interventions. As evidenced by the distinct postural strategies observed across different populations—ranging from children with ADHD to community-dwelling older adults and stroke survivors—future therapeutic and training programs must be meticulously tailored to an individual's specific demographic, cognitive, and neurological profile.

Second, the complex role of visuomotor integration demands practical, real-world translation. While targeted visual feedback can successfully enhance inter-limb coordination, an over-reliance on immediate visual cues can simultaneously provoke maladaptive, high-risk motor behaviors, such as spin turns in older adults. Future studies should emphasize the development of multisensory training paradigms that optimize sensory reweighting in dynamic, unpredictable daily environments, ensuring that sensory priorities align with biomechanical safety.

Finally, the clinical implementation of advanced biomechanical and computational methodologies will be paramount. Transitioning sophisticated tools, such as perturbation-based mathematical modeling and direct CoP modification techniques, from the laboratory to routine clinical practice may substantially improve our ability to objectively assess and potentially predict fall risk and maximize the efficacy of targeted rehabilitation.

